# Maternal Big Five personality traits and breastfeeding outcomes: what we know and what we don’t know

**DOI:** 10.3389/fpubh.2024.1484547

**Published:** 2024-11-29

**Authors:** Donata Bessey

**Affiliations:** EastAsia International College, Yonsei University, Wonju, Republic of Korea

**Keywords:** breastfeeding, lactation, personality traits, Big Five, HEXACO

## Abstract

**Introduction:**

Exclusive breastfeeding—feeding an infant only breast milk for the first 6 months of life—is recognized as the preventive intervention with the greatest potential to reduce child mortality. However, the World Health Organization (WHO) estimates that only 44% of all infants globally are exclusively breastfed for the first 6 months of life. Research into the barriers to meeting this goal of exclusive breastfeeding suggests an important role for sociodemographic factors. Maternal personality traits, another possible factor affecting infant feeding outcomes, have received relatively sparse attention from researchers and are the focus of this mini-review.

**Methods:**

Three databases and one peer-reviewed journal in lactation that was not included in either were systematically searched. Studies that analyzed the relationship between maternal Big Five personality traits and breastfeeding or lactation outcomes were included in this mini-review. In addition, the reference sections of all included studies were searched for other possible matches, resulting in one more study being included.

**Results:**

Eleven studies dating from 2006 to 2022 met the criteria for inclusion in this mini-review. In total, they included *n* = 19,425 participants. Due to the differences in methodology, statistical analysis, and breastfeeding outcomes analyzed, they were summarized using a narrative synthesis.

**Conclusion:**

There were no emerging patterns regarding associations between Big Five personality traits and breastfeeding outcomes. While personality traits may play a role, their influence might be moderated by other factors, including other psychological, social, and demographic variables. More studies employing state-of-the-art research design and analysis methods are needed to see whether patterns will emerge.

## 1 Introduction

Exclusive breastfeeding for the first 6 months of life is the preventive intervention with the greatest potential to reduce child mortality (1). Optimal breastfeeding practices also include initiation within the first hour of life and continuing to breastfeed for up to 2 years or longer as desired by the mother and infant, in addition to introducing complementary foods at around 6 months of age. Breastfeeding is protective against respiratory infections (2, 3), gastrointestinal infections and diarrhea (2, 3), acute otitis media (3–5), sudden infant death syndrome (SIDS) (3, 6, 7), and necrotizing enterocolitis (NEC) in preterm infants (8). It may be associated with a decrease in the infant’s risk for other health conditions (9). In addition, it is also associated with a lower risk of developing breast cancer (3, 10), ovarian, and endometrial cancer (3, 10–12), and cardiovascular disease (13) for the lactating mother, in addition to protection against other diseases, such as type 2 diabetes (3, 14–16). Therefore, breastfeeding is a unique health behavior that substantially benefits both parent and infant. However, despite those well-documented numerous benefits, the World Health Organization (WHO) estimates that only 44% of all infants worldwide are exclusively breastfed for the first 6 months of life (17). Research into the barriers to meeting this goal of exclusive breastfeeding underlines the importance of sociodemographic factors, according to a recent meta-review (18), which found evidence for adverse effects of young maternal age, a low level of education, having to return to work within 12 weeks postpartum, having birthed via cesarean section, and inadequate milk supply. Maternal personality traits, another possible factor affecting infant feeding outcomes, have received little attention from researchers. This mini-review explores this under-researched topic and investigates the possible impact of maternal personality traits on infant feeding outcomes.

Personality traits are enduring patterns of thoughts, feelings, and behaviors that show stability and change over time (19). They tend to change toward greater maturity over the lifespan, with important interpersonal differences in the timing and direction of those changes (20). However, the stability of personality traits is debated, with some researchers arguing that stability depends on environmental factors and measurement methods (21). Personality traits differ from other constructs like mood, which are more transient and contribute to variance in affective measures (22).

The Big Five model, encompassing Openness, Conscientiousness, Extraversion, Agreeableness, and Neuroticism (Emotional Stability), is based on language descriptors and defines human personality at the broadest level (23, 24). Openness to Experience is characterized by traits such as imagination, curiosity, and creativity, contrasted with shallowness and imperceptiveness. Conscientiousness involves traits such as organization, thoroughness, and reliability versus carelessness, negligence, and unreliability. Extraversion encompasses traits such as talkativeness, assertiveness, and high activity levels, as opposed to silence, passivity, and reserve. Agreeableness contrasts traits like kindness, trust, and warmth with hostility, selfishness, and distrust. Lastly, Emotional Stability includes traits like nervousness, moodiness, and temperamentality, as opposed to calmness, self-confidence, and emotional consistency (24). The model was chosen due to its comprehensive framework, and it has been extensively validated across different cultures and populations, making it a robust tool for psychological research (25) which is particularly useful in research areas such as health behaviors, where various personality dimensions can influence outcomes differently (26). For instance, Conscientiousness has been linked to health-promoting behaviors (27), while Neuroticism often correlates with negative health outcomes (28). Conscientiousness is often linked to health-promoting behaviors through mechanisms like self-discipline and goal-directed behavior. Neuroticism, on the other hand, might be associated with negative outcomes due to emotional instability, which can increase stress and hinder adaptive coping strategies. Extraversion may facilitate better social support networks, while Openness is associated with a greater willingness to engage in novel and exploratory behaviors. Finally, Agreeableness could promote positive social interactions, leading to healthier behaviors through cooperation and conflict avoidance.

Its broad applicability makes the model a good choice in examining health behaviors like breastfeeding, which multiple personality dimensions can simultaneously influence. In contrast, specific traits like negative affectivity or trait anxiety, while important, offer a narrower focus and might not capture the full range of personality influences on breastfeeding outcomes.

## 2 Methods

From July 10 to July 12, 2024, the following databases were searched: the Web of Science Core Collection, PubMed, and ProQuest Psychology (which includes APA PsycArticles. APA PsycInfo, APA PsycTests®, Psychology Database, and PTSDpubs). The search expression used was (“maternal personality” OR “maternal psychology” OR “maternal temperament” OR “parental personality” OR “parental psychology” OR “parental temperament” OR “mother’s personality” OR “mother’s psychology” OR “mother’s temperament” OR “parent’s personality” OR “parent’s psychology” OR “parent’s temperament” OR “personality traits” OR “Big Five”) AND (“breastfeeding initiation” OR “exclusive breastfeeding” OR “breastfeeding duration” OR “breastfeeding outcome” OR “breastfeeding behavior” OR “breastfeeding success” OR “breastfeeding practices” OR “lactation initiation” OR “lactation duration” OR “lactation outcome” OR “lactation success” OR “lactation practices” OR “lactation behavior”). This yielded 26 results in the Web of Science, 1802 in PubMed, and 128 in ProQuest Psychology. After analyzing the abstract and, if needed, the body of the article, 10 results from the Web of Science, 12 from PubMed, and five results from ProQuest Psychology met the criteria for inclusion (i.e., they analyzed the associations between maternal personality traits and breastfeeding outcomes). Since “Clinical Lactation,” another professional research journal in the field, is not included in PubMed, a separate search using the same search expression was conducted on July 12, 2024; no additional articles were found. After checking for duplicates, 13 studies remained. However, one study used the Lüscher Color Test as a personality trait measure. It is included in a published list of discredited procedures in psychology (63), and therefore, the study was not included in this mini-review. Two further studies that used the same data set for analysis employed a fundamentally flawed methodology: the sample consisted only of mothers who had exclusively breastfed, eliminating any variability in breastfeeding status. This lack of variation precludes any meaningful analysis of factors influencing the choice to breastfeed exclusively, as all participants have already made the same decision, thereby violating a basic principle of research design, which requires variability in the dependent variable for analysis. In addition, the scale used as the dependent variable is mischaracterized because instead of measuring actual breastfeeding outcomes or behaviors, it comprises subjective items that do not reflect exclusive breastfeeding practices. This misalignment between the intended measurement and the actual construct assessed further invalidates the findings (29). Finally, the studies used data on breastfeeding intentions and personality traits collected retrospectively, 6 to 12 months postpartum, introducing significant recall bias and confounding factors, as the mothers’ breastfeeding decisions and experiences have already been made and could influence their responses. These design flaws led to excluding the two studies from this mini-review.

An additional search of the references in all of the included studies resulted in one more article that was included (30), resulting in a total of 11 studies.

## 3 Results

The 11 studies were conducted in Chile, Croatia (two), Germany, Italy, the Netherlands, Poland, Spain, Türkiye, the United Kingdom, and the United States. All can be classified as primary, observational research in epidemiology (31). Table 1 provides an overview and summary.

**Table 1:** Overview of the studies included in this mini-review.

Nr	Study location	Design	Sample size	Big Five inventory used	Outcome variable(s)	Statistical analysis method(s)	Results
1	Wagner et al. (42) USA	prospective study	*n* = 81	NEO Personality Inventory-Revised [NEO-PI-R], 264 items	Initiation	Comparison of personality traits scores between mothers who exclusively breastfed and exclusively formula-fed throughout postpartum hospital stay (t-tests), multivariate logit for initiation (control variables: infant health, ethnicity, income-related variables)	t-tests: those who initiated breastfeeding score higher on Agreeableness, Extraversion, and Openness Multivariate logits: Extraversion and Openness remain statistically significant
2	Brown (43) UK	cross-sectional survey	*n* = 602	Big Five Ten-Item Personality Measure (66)	Initiation Any breastfeeding at birth and at 2, 6, 12, and 26 weeks postpartum	Comparison of personality traits scores between mothers with any breastfeeding and with exclusive formula feeding (MANCOVA)	Those with any breastfeeding score higher on Extraversion at birth, 2, and 6 weeks Those with any breastfeeding score higher on Emotional Stability (Neuroticism) at birth and all time points postpartum Those with any breastfeeding score higher on Conscientiousness at birth only
3	Di Mattei et al. (37) Italy	cross-sectional survey	*n* = 160	*Big Five Inventory* (BFI) (67) (44 items)	Intention to breastfeed as measured by score on Iowa Infant Feeding Attitude Scale (32)	Correlation coefficients between traits and IIFAS score, multivariate ordinary least squares regression (controls: age, self-employed status, parity, fed formula as infant)	Significant negative correlation between Neuroticism and IIFAS score Persists in multiple regression
4	Imširagić et al. (71) Croatia	prospective cohort study	*n* = 259	Big Five Inventory (57) (44 items)	Exclusive breastfeeding 6 to 8 weeks after birth	Bivariate and multivariate logistic regression, odds ratios reported Control variables: age, employment, parity, mode of delivery, PTSD (62), depression EPDS (60)	Significant negative association between Neuroticism and exclusive breastfeeding Disappears in multiple regression
5	Keller et al. (39) Croatia	observational cross-sectional study	*n* = 303	Thompson’s International English Big-Five Mini-Markers (40 items) (58)	Duration of any breastfeeding, any breastfeeding for ≥6 months (including feeding expressed milk)	Correlation coefficients for duration, bivariate logistic regression for breastfeeding for ≥6 months	Duration: Significantly positive correlations with Agreeableness and Openness, significantly negative correlation with Neuroticism Breastfeeding for ≥6 months: no relationship
6	Catala et al. (45) (Spain)	longitudinal prospective study	*n* = 116	Big Five NEO-FFI (60 items)	Initiation <1 h of birth, breastfeeding on demand at 4 months postpartum	Comparison of personality traits scores between mothers who initiated <1 h of birth/breastfed on demand at 4 months and those who did not (t-tests)	Initiation: No differences found in t-tests Those who continued to breastfeed on demand at 4 months scored higher on Openness
7	Maliszewska et al. (30) Poland	case-cohort study	*n* = 251	Big Five NEO-FFI (60 items)	“Almost exclusive” breastfeeding (currently exclusive, with possible formula supplementation before)/any breastfeeding at 6 months postpartum	t-tests, multivariate logit regressions Controls: maternal habitation; education; marriage; risk for postpartum depression at 1 week and 3 months (EPDS >12 or self-harm) (60); maternal professional activity; mode of delivery; parity; any breastfeeding within the first week; social support (61)	No differences when comparing “almost exclusive” to any breastfeeding in t-tests No statistically significant associations in multivariate regressions (Openness at 10%)
8	Farkas & Girard (38) Chile	prospective cohort study (Encuesta Longitudinal de la Primera Infancia)	*n* = 13,738	Big Five inventory (44 items), Casullo (59)	initiation, duration (exclusive/mixed)	Fisher’s exact test/Chi-square-test for differences between those who did vs. did not initiate breastfeeding after birth Multivariate logit for initiation, multivariate OLS for duration Controls: place of residence, marital status, maternal socio-economic background, age, maternal and infant risk factors, delivery mode, infant sex, maternal cognitive ability	Those who did not initiate score higher on Neuroticism, disappears in regression Ordinary least squares regression: low Neuroticism is significantly associated with longer duration
9	Verbeek et al. (40) Netherlands	population-based prospective cohort study	*n* = 2,927	Big Five NEO-FFI (60 items)	6 months of exclusive breastfeeding (WHO definition)	Multivariate logit regressions Controls: age, relationship status, parity, and SES Use imputation technique for missing values	Odds ratios reported: Agreeableness +Extraversion –Neuroticism –Openness +Association for neuroticism disappears when controlling for anxiety and when controlling for depression
10	Ludwig et al. (41) Germany	Birth cohort study	*n* = 780	Big Five Inventory (BFI-10) (68), 10 items	4 months/6 months of exclusive breastfeeding (including water/tea)	Wilcoxon rank-sum tests for differences between those who did and did not exclusively breastfeed for 4/6 months Multivariate logit regressions Controls: predictor variables that were significantly different in tests	Lower score of Neuroticism, higher score of Openness for exclusive breastfeeders No statistically significant associations with Big Five traits in multivariate logit regressions
11	Sercekus et al. (44) Turkey	Cross-sectional study	*n* = 208	Big Five Ten-Item Personality Measure (66)	LATCH score (33)	Kruskal-Wallis tests, groups defined by the highest-scoring personality trait	Differences between groups are statistically significant; LATCH scores highest in those with Extraversion being the highest-scoring trait, lowest in those with Agreeableness being the highest-scoring trait

Regarding breastfeeding outcomes, attitudes toward breastfeeding (measured using the Iowa Infant Feeding Attitudes Scale (IIFAS), (32)), breastfeeding quality measured using the LATCH score [a tool to assess the quality of the infant’s latch or attachment to the breast, (33)], breastfeeding duration, and any/exclusive/on-demand breastfeeding at certain ages (such as 4 or 6 months postpartum) were used; some studies also included expressing milk using a pump as breastfeeding. The study on attitudes was included because research using the Theory of Planned Behavior (34) approach has suggested that infant feeding decisions are often made during pregnancy (35, 64), with attitudes and knowledge about breastfeeding influencing both decisions and feeding outcomes [see, for example, Lawton et al. (69), Naja et al. (70)] (36).

In addition, the research methodology and statistical analysis methods used differed widely, from cross-sectional to retrospective and prospective (population) cohort studies for the methodology and classical hypothesis testing or bivariate/multivariate regression used for the statistical analysis. Lastly, the resulting sample sizes also varied greatly, ranging from *n* = 87 in an early study to *n* = 13,738 in a population-based cohort study. Therefore, the following section offers a narrative synthesis of the results by personality traits and in chronological order of outcomes through pregnancy, the postpartum hospital stay, and the first months of life of the infant. More detailed information on the included studies, including methodology, is provided in Table 1.

Regarding Big Five Neuroticism, the following studies reported statistically significant associations with breastfeeding intentions and outcomes. Di Mattei et al. (37) found a negative correlation between Neuroticism and attitudes toward breastfeeding, as measured by IIFAS scores during pregnancy. Several studies report negative bivariate correlations between Neuroticism and breastfeeding outcomes that disappear in multivariate analysis: Farkas and Gisrard (38) report that those who did not initiate breastfeeding after birth score higher on Neuroticism, but the association disappears in multivariate regressions. Similarly, Srkalović Imširagić et al. (71) report a negative association between Neuroticism and exclusive breastfeeding at 6 to 8 weeks postpartum that disappears in multivariate regressions. For any breastfeeding ≥6 months, Keller et al. (39) report a significant negative correlation with Neuroticism that disappears in multivariate regression, and Verbeek et al. (40) report a negative association between Neuroticism and reaching the WHO’s 6-month exclusive breastfeeding recommendation. Still, this effect disappears when controlling for depression and anxiety. Similarly, Ludwig et al. (41) found a significant negative association between neuroticism and reaching the WHO goal, which disappeared in multivariate regression. Lastly, Farkas and Girard (38) report that lower Neuroticism (higher emotional stability) is associated with longer breastfeeding duration.

For Big Five Extraversion, both Wagner et al. (42) and Brown (43) report a statistically significant correlation between Extraversion and breastfeeding initiation, and Sercekus et al. (44) found significantly higher LATCH scores for mothers who score highest on the Extraversion trait. Brown (43) also reported that those who reported any breastfeeding at birth, 2, and 6 weeks postpartum scored higher on Extraversion than those who stopped breastfeeding. However, Verbeek et al. (40) found a significant negative association between the trait and meeting the WHO’s 6-month exclusive breastfeeding recommendation, while Ludwig et al. (41) as well as Maliszewska et al. (30) reported no significant association.

Concerning Big Five Openness, Wagner et al. (42) also reported a statistically significant positive correlation between the trait and breastfeeding initiation. Catala et al. (45) reported higher Openness scores for those continuing breastfeeding on demand at 4 months postpartum. Keller et al. (39) found a significant positive correlation between Openness and duration of any breastfeeding ≥6 months in bivariate regressions, and Verbeek et al. (40) reported a positive association between Openness and 6 months of exclusive breastfeeding. However, Ludwig et al. (41) and Maliszewska et al. (30)found no significant association between Openness and the same outcome.

For Big Five agreeableness, Wagner et al. (42) reported a significant positive correlation with initiation, which disappeared when controlling for infant health, ethnicity, and income. Keller et al. (39) found a significant positive correlation with any breastfeeding for ≥6 months, and Verbeek et al. (40) found a significant positive association with meeting the goal of 6 months of exclusive breastfeeding. Again, Ludwig et al. (41) and Maliszewska et al. (30)found no significant association between Agreeableness and the same outcome.

Lastly, for Big Five Conscientiousness, Brown (43) reported significantly higher Conscientiousness scores for those with any breastfeeding at birth but no significant differences at later time points. No other studies reported any significant associations.

## 4 Discussion

The 11 studies included in this mini-review present highly divergent results regarding the relationship between Big Five personality traits and breastfeeding outcomes. The studies varied significantly in methodologies, including cross-sectional and cohort studies, using convenience and representative samples. They used different tools to measure breastfeeding outcomes, such as the Iowa Infant Feeding Attitudes Scale (IIFAS), LATCH scores, and breastfeeding duration and exclusivity at various postpartum stages. Consistent patterns were hard to identify. Some studies reported significant correlations between specific personality traits and breastfeeding outcomes in bivariate analyses. Still, these associations often disappeared in multivariate regressions when controlling for other factors, suggesting that Big Five personality traits might not independently predict breastfeeding behaviors. Additionally, the results were mixed when examining the duration and exclusivity of breastfeeding: while some studies reported associations with a certain trait, others found no significant links with the same trait. This variability in findings underscores the complexity of breastfeeding outcomes and their determinants. While personality traits may play a role, their influence is likely moderated by other factors, including other psychological, social, and demographic variables. More studies employing state-of-the-art research design and analysis methods are needed to see whether patterns will emerge. In addition, most of the studies had exclusion criteria that effectively limited participation to low obstetric risk patients, so research on other populations, such as high-risk parents or infants, is needed.

The results suggest contrasts with results on other health behaviors and their correlations with Big Five personality traits: according to a meta-synthesis of results, the associations tend to be stronger for Agreeableness, Conscientiousness, and Neuroticism than for Extraversion or Openness to experience (46). Following the model proposed by De Young (47), which suggests that Big Five traits represent two higher-order factors, stability and plasticity, traits related to stability (Agreeableness, Conscientiousness, and Neuroticism) seem to be more important than those related to plasticity (Extraversion and Openness). In addition, they tend to be strongest for mental health outcomes, followed by health behaviors (such as smoking) and physical health outcomes (46). Pletzer et al. (48) provide a meta-synthesis of the HEXACO personality traits model (which, among other differences, adds a sixth trait, honesty-humility) and correlations with health-promoting behaviors. In contrast to Strickhouser et al. (46), they reported the strongest correlations with Conscientiousness, followed by Extraversion, Openness, Honesty-Humility, and Agreeableness, but none with Emotionality. Both meta-analyses also reported substantial variations in correlation strength between traits and specific health behaviors. While breastfeeding should be considered a preventive health behavior in the sense that it requires sustained efforts and directly affects both maternal and infant health outcomes, as discussed in the introduction, research also suggests that the artificial infant milk industry’s predatory marketing efforts (49) are not sufficiently regulated by government policies (50) and directly undermine breastfeeding through several channels, including false claims that “position formula as close to, equivalent or superior to breast milk despite growing evidence that breast milk and breastfeeding have unique properties that cannot be replicated by artificial formula” (51). More recently, the industry skillfully employs online marketing strategies (52), often in violation of the International Code of Marketing of Breast-milk Substitutes 1981 (53), that may directly affect parents’ feeding choices (54).

Since breastfeeding promotes the health of both parent and child, future research could focus more on facets of personality traits specifically related to prosocial behavior, especially altruism. Candidates might include facets of the Big Five traits (55), such as altruism or sympathy, as facets of Agreeableness, or empathy and sensitivity as facets of Emotionality in the HEXACO model. The honesty-humility trait in the abovementioned HEXACO model might be another candidate since individuals scoring high on the trait are more likely to engage in altruistic behaviors, making it a strong predictor of prosocial actions, in addition to Agreeableness and Emotionality (56).

While the studies reviewed rely on two-sample hypothesis testing, correlations, and single or multiple regression analyses to explore the relationship between maternal personality traits and breastfeeding outcomes, these methods may not fully capture the complexity of the underlying processes since personality traits might affect beliefs about ability to breastfeed, measured by constructs such as breastfeeding self-efficacy (72), which might, in turn, affect breastfeeding outcomes. Such indirect relationships might be better understood through more sophisticated analytical techniques, such as mediation or moderation analyses or structural equation modeling (SEM). Four-way causal mediation analysis (65) could be applied to examine how maternal personality traits influence breastfeeding outcomes, distinguishing between direct effects, mediated effects via constructs such as breastfeeding efficacy, and any interaction effects between personality traits and other constructs. This approach offers a detailed breakdown of potential causal mechanisms, allowing for a better understanding of how these factors collectively shape breastfeeding behaviors. By using these methods, future research could provide a more detailed understanding of how maternal personality traits influence breastfeeding, potentially leading to more targeted and effective interventions.

## References

[1] Jones G Steketee RW Black RE Bhutta ZA Morris SS Bellagio Child Survival Study Group. How many child deaths can we prevent this year? Lancet. (2003) 362:65–71. doi: 10.1016/S0140-6736(03)13811-1 12853204

[2] Horta BL Victora CG World Health Organization. Short-term effects of breastfeeding: A systematic review on the benefits of breastfeeding on diarrhoea and pneumonia mortality. Geneva: WHO (2013).

[3] Ip S Chung M Raman G Chew P Magula N DeVine D. Breastfeeding and maternal and infant health outcomes in developed countries. Evidence report/technology assessment no. 153. Rockville, MD: Agency for Healthcare Research and Quality (2007) PMC4781366 17764214

[4] Bowatte G Tham R Allen KJ Tan DJ Lau MXZ Dai X. Breastfeeding and childhood acute otitis media: a systematic review and meta-analysis. Acta Paediatr. (2015) 104:85–95. doi: 10.1111/apa.13151, PMID: 26265016

[5] Tenenbaum Weiss Y Ovnat Tamir S Globus O Marom T. Protective characteristics of human breast milk on early childhood otitis media: a narrative review. Breastfeed Med. (2024) 19:73–80. doi: 10.1089/bfm.2023.0237, PMID: 38386988

[6] Hauck FR Thompson JM Tanabe KO Moon RY Vennemann MM. Breastfeeding and reduced risk of sudden infant death syndrome: a meta-analysis. Pediatrics. (2011) 128:103–10. doi: 10.1542/peds.2010-3000, PMID: 21669892

[7] Thompson J Tanabe K Moon RY Mitchell EA McGarvey C Tappin D. Duration of breastfeeding and risk of SIDS: an individual participant data meta-analysis. Pediatrics. (2017) 140:e20171147. doi: 10.1542/peds.2017-1147 29084835

[8] Quigley M Embleton ND McGuire W. Formula versus donor breast milk for feeding preterm or low birth weight infants. Cochrane Database Syst Rev. (2019) 7:CD002971. doi: 10.1002/14651858.CD002971.pub5, PMID: 31322731 PMC6640412

[9] Horta BL Victora CG World Health Organization. Long-term effects of breastfeeding: A systematic review. Geneva: World Health Organization (2013).

[10] Chowdhury R Sinha B Sankar MJ Taneja S Bhandari N Rollins N. Breastfeeding and maternal health outcomes: a systematic review and meta-analysis. Acta Paediatr. (2015) 104:96–113. doi: 10.1111/apa.13102, PMID: 26172878 PMC4670483

[11] Luan NN Wu QJ Gong TT Vogtmann E Wang YL Lin B. Breastfeeding and ovarian cancer risk: a meta-analysis of epidemiologic studies. Am J Clin Nutr. (2013) 98:1020–31. doi: 10.3945/ajcn.113.062794, PMID: 23966430 PMC3778857

[12] Sung HK Ma SH Choi JY Hwang Y Ahn C Kim BG. The effect of breastfeeding duration and parity on the risk of epithelial ovarian cancer: a systematic review and meta-analysis. J Prev Med Public Health. (2016) 49:349–66. doi: 10.3961/jpmph.16.066, PMID: 27951628 PMC5160134

[13] Tschiderer L Seekircher L Kunutsor SK Peters SA O’Keeffe LM Willeit P. Breastfeeding is associated with a reduced maternal cardiovascular risk: systematic review and meta-analysis involving data from 8 studies and 1,192,700 parous women. J Am Heart Assoc. (2022) 11:e022746. doi: 10.1161/JAHA.121.022746, PMID: 35014854 PMC9238515

[14] Aune D Norat T Romundstad P Vatten LJ. Breastfeeding and the maternal risk of type 2 diabetes: a systematic review and dose-response meta-analysis of cohort studies. Nutr Metab Cardiovasc Dis. (2014) 24:107–15. doi: 10.1016/j.numecd.2013.10.028, PMID: 24439841

[15] Jäger S Jacobs S Kröger J Fritsche A Schienkiewitz A Rubin D. Breastfeeding and maternal risk of type 2 diabetes: a prospective study and meta-analysis. Diabetologia. (2014) 57:1355–65. doi: 10.1007/s00125-014-3247-3, PMID: 24789344 PMC4052010

[16] Pinho-Gomes AC Morelli G Jones A Woodward M. Association of lactation with maternal risk of type 2 diabetes: a systematic review and meta-analysis of observational studies. Diabetes Obes Metab. (2021) 23:1902–16. doi: 10.1111/dom.14417, PMID: 33908692

[17] World Health Organization. Infant and young child feeding. Geneva: World Health Organization (2023).

[18] Mangrio E Persson K Bramhagen AC. Sociodemographic, physical, mental and social factors in the cessation of breastfeeding before 6 months: a systematic review. Scand J Caring Sci. (2018) 32:451–65. doi: 10.1111/scs.12489, PMID: 28569436

[19] Hampson S Goldberg LR. Personality stability and change over time. The wiley encyclopedia of personality and individual differences: models and theories. eds. Bernardo. J. Carducci, Christopher S. Nave, Jeffrey S. Mio, Ronald E. Riggio. John Wiley & Sons, Hoboken: NJ. (2020).

[20] Bleidorn W Hopwood CJ Back MD Denissen JJ Hennecke M Hill PL. Personality trait stability and change. Perspect Sci. (2021) 2:e6009. doi: 10.5964/ps.6009

[21] Ardelt M. Still stable after all these years? Personality stability theory revisited. Soc Psychol Q. (2000) 63:392–405. doi: 10.2307/2695848

[22] Anusic I Schimmack U. Stability and change of personality traits, self-esteem, and well-being: introducing the meta-analytic stability and change model of retest correlations. J Pers Soc Psychol. (2016) 110:766–81. doi: 10.1037/pspp0000066, PMID: 26619304

[23] Goldberg LR. A historical survey of personality scales and inventories In: McReynolds P, editor. Advances in psychological assessment. Palo Alto, CA: Science and Behavior Books (1971). 293–336.

[24] Goldberg LR. The structure of phenotypic personality traits. Am Psychol. (1993) 48:26–34. doi: 10.1037/0003-066X.48.1.26 8427480

[25] McCrae RR Costa PT Jr. The five-factor theory of personality In: John OP Robins RW Pervin LA, editors. Handbook of personality: Theory and research. 3rd ed. New York: Guilford Press (2008). 159–81.

[26] John OP Naumann LP Soto CJ. Paradigm shift to the integrative big five trait taxonomy: history, measurement, and conceptual issues In: John OP Robins RW Pervin LA, editors. Handbook of personality: Theory and research. 3rd ed. New York: Guilford Press (2008). 114–58.

[27] Bogg T Roberts BW. The case for conscientiousness: evidence and implications for a personality trait marker of health and longevity. Ann Behav Med. (2013) 45:278–88. doi: 10.1007/s12160-012-9454-6, PMID: 23225322 PMC3604184

[28] Smith TW. Personality as risk and resilience in physical health. Curr Dir Psychol Sci. (2006) 15:227–31. doi: 10.1111/j.1467-8721.2006.00441.x

[29] DeVellis RF. Scale development: Theory and applications. 4th ed SAGE Publications (2016). California: Thousand Oaks.

[30] Maliszewska KM Bidzan M Świątkowska-Freund M Preis K. Socio-demographic and psychological determinants of exclusive breastfeeding after six months postpartum—a polish case-cohort study. Ginekol Pol. (2018) 89:153–9. doi: 10.5603/GP.a2018.0026, PMID: 29664551

[31] Röhrig B Du Prel JB Wachtlin D Blettner M. Types of study in medical research: part 3 of a series on evaluation of scientific publications. Dtsch Arztebl Int. (2009) 106:262–8. doi: 10.3238/arztebl.2009.0262, PMID: 19547627 PMC2689572

[32] De la Mora A Russell D Dungy C Losch M Dusdieker L. The Iowa infant feeding attitude scale: analysis of reliability and validity. J Appl Soc Psychol. (1999) 29:2362–80. doi: 10.1111/j.1559-1816.1999.tb00115.x

[33] Jensen D Wallace S Kelsay P. LATCH: a breastfeeding charting system and documentation tool. J Obstet Gynecol Neonatal Nurs. (1994) 23:27–32. doi: 10.1111/j.1552-6909.1994.tb01847.x 8176525

[34] Ajzen I. The theory of planned behavior. Organ Behav Hum Decis Process. (1991) 50:179–211. doi: 10.1016/0749-5978(91)90020-T

[35] Shaker I Scott JA Reid M. Infant feeding attitudes of expectant parents: breastfeeding and formula feeding. J Adv Nurs. (2004) 45:260–8. doi: 10.1046/j.1365-2648.2003.02887.x 14720243

[36] Kornides M Kitsantas P. Evaluation of breastfeeding promotion, support, and knowledge of benefits on breastfeeding outcomes. J Child Health Care. (2013) 17:264–73. doi: 10.1177/1367493512461460, PMID: 23439591 PMC4086458

[37] Di Mattei VE Carnelli L Bernardi M Jongerius C Brombin C Cugnata F. Identification of socio-demographic and psychological factors affecting women’s propensity to breastfeed: an Italian cohort. Front Psychol. (2016) 7:1872. doi: 10.3389/fpsyg.2016.01872, PMID: 27965610 PMC5126723

[38] Farkas C Girard LC. Breastfeeding initiation and duration in Chile: understanding the social and health determinants. J Epidemiol Community Health. (2019) 73:637–44. doi: 10.1136/jech-2018-211148, PMID: 30867222

[39] Keller N Medved V Armano G. The influence of maternal personality and risk factors for impaired mother-infant bonding on breastfeeding duration. Breastfeed Med. (2016) 11:532–7. doi: 10.1089/bfm.2016.0093, PMID: 27805427

[40] Verbeek T Quittner L de Cock P de Groot N Bockting CL Burger H. Personality traits predict meeting the WHO recommendation of 6 months' breastfeeding: a prospective general population cohort study. Adv Neonatal Care. (2019) 19:118–26. doi: 10.1097/ANC.0000000000000547, PMID: 30325749

[41] Ludwig A Doyle IM Löffler A Breckenkamp J Spallek J Razum O. The impact of psychosocial factors on breastfeeding duration in the BaBi-study. Analysis of a birth cohort study in Germany. Midwifery. (2020) 86:102688. doi: 10.1016/j.midw.2020.102688, PMID: 32276156

[42] Wagner CL Wagner MT Ebeling M Chatman KG Cohen M Hulsey TC. The role of personality and other factors in a mother’s decision to initiate breastfeeding. J Hum Lact. (2006) 22:16–26. doi: 10.1177/0890334405283624 16467284

[43] Brown A. Maternal trait personality and breastfeeding duration: the importance of confidence and social support. J Adv Nurs. (2014) 70:587–98. doi: 10.1111/jan.12219, PMID: 23919294 PMC4114133

[44] Sercekus P Ozan Y Yenal K. The relationship between breastfeeding success and maternal personality traits. J Nurs Midwifery Sci. (2022) 9:12–21. doi: 10.4103/jnms.jnms_16_21

[45] Catala P Peñacoba C Carmona J Marin D. Maternal personality and psychosocial variables associated with initiation compared to maintenance of breastfeeding: a study in low obstetric risk women. Breastfeed Med. (2018) 13:680–6. doi: 10.1089/bfm.2018.0034, PMID: 30403497

[46] Strickhouser JE Zell E Krizan Z. Does personality predict health and well-being? A metasynthesis Health Psychol. (2017) 36:797–810. doi: 10.1037/hea0000475, PMID: 28277701

[47] DeYoung CG. Higher-order factors of the big five in a multi-informant sample. J Pers Soc Psychol. (2006) 91:1138–51. doi: 10.1037/0022-3514.91.6.1138, PMID: 17144770

[48] Pletzer JL Thielmann I Zettler I. Who is healthier? A meta-analysis of the relations between the HEXACO personality domains and health outcomes. Eur J Personal. (2024) 38:342–64. doi: 10.1177/08902070231174574

[49] Rollins N Piwoz E Baker P Kingston G Mabaso KM McCoy D. Marketing of commercial milk formula: a system to capture parents, communities, science, and policy. Lancet. (2023) 401:486–502. doi: 10.1016/S0140-6736(22)01931-6, PMID: 36764314

[50] Baker P Smith JP Garde A Grummer-Strawn LM Wood B Sen G. The political economy of infant and young child feeding: confronting corporate power, overcoming structural barriers, and accelerating progress. Lancet. (2023) 401:503–24. doi: 10.1016/S0140-6736(22)01933-X 36764315

[51] World Health Organization. How the marketing of formula milk influences our decisions on infant feeding. Geneva: World Health Organization (2022).

[52] Jones A Bhaumik S Morelli G Zhao J Hendry M Grummer-Strawn L. Digital marketing of breast-milk substitutes: a systematic scoping review. Curr Nutr Rep. (2022) 11:416–30. doi: 10.1007/s13668-022-00414-3, PMID: 35507274 PMC9066994

[53] World Health Organization. International code of the marketing of breast-milk substitutes. Geneva: World Health Organization (1981).

[54] Unar-Munguía M Santos-Guzmán A Mota-Castillo PJ Ceballos-Rasgado M Tolentino-Mayo L Aguilera MS. Digital marketing of formula and baby food negatively influences breastfeeding and complementary feeding: a cross-sectional study and video recording of parental exposure in Mexico. BMJ Glob Health. (2022) 7:e009904. doi: 10.1136/bmjgh-2022-009904 PMC9594510 36343968

[55] Paunonen SV Ashton MC. Big five factors and facets and the prediction of behavior. J Pers Soc Psychol. (2001) 81:524–39. doi: 10.1037/0022-3514.81.3.524 11554651

[56] Ashton MC Lee K. Empirical, theoretical, and practical advantages of the HEXACO model of personality structure. Personal Soc Psychol Rev. (2007) 11:150–66. doi: 10.1177/1088868306294907, PMID: 18453460

[57] Benet-Martínez V John OP. Los Cinco Grandes across cultures and ethnic groups: multitrait multimethod analyses of the big five in Spanish and English. J Pers Soc Psychol. (1998) 75:729–50. doi: 10.1037/0022-3514.75.3.729, PMID: 9781409

[58] Thompson ER. Development and validation of an international English big-five mini-markers. Personal Individ Differ. (2008) 45:542–8. doi: 10.1016/j.paid.2008.06.013

[59] Casullo M. Big five inventory. Buenos Aires, Argentina: Universidad de Buenos Aires (2000).

[60] Cox JL Holden JM Sagovsky R. Detection of postnatal depression: development of the 10-item Edinburgh postnatal depression scale. Br J Psychiatry. (1987) 150:782–6. doi: 10.1192/bjp.150.6.782 3651732

[61] Schwarzer R Schulz U. The Berlin social support scales (BSSS): assessment of social support in coping with stress In: Sarason IG Sarason BR Pierce GR, editors. Social support: An interactional view. New York: Wiley (2000). 43–67.

[62] Weiss DS Marmar CR. The impact of event scale-revised In: Wilson JP Keane TM, editors. Assessing psychological trauma and PTSD: A Practitioner’s handbook. New York: Guilford Press (1997). 399–411.

[63] Koocher G McMann M Stout A Norcross J. Discredited Assessment and Treatment Methods Used with Children and Adolescents: A Delphi Poll. J. Clin. Child Adolesc. Psychol. (2014) 44:722–729. 24766155 10.1080/15374416.2014.895941

[64] Swanson V Power KG. Initiation and continuation of breastfeeding: Theory of planned behaviour. J. Adv. Nurs. (2005) 50:272–282. doi: 10.1111/j.1365-2648.2005.03390.x 15811106

[65] DiScacciati A Bellavia A Orsini N Greenland S. Statistical methods for mediation analysis with exposure-mediator interactions. Am J Epidemiol, (2019) 188:879–889. doi: 10.1093/aje/kwz002

[66] Gosling SD Rentfrow PJ Swann WB. A very brief measure of the Big-Five personality domains. J. Res. Pers. (2003) 37:504–528. doi: 10.1016/S0092-6566(03)00046-1

[67] John OP Donahue EM Kentle RL. The Big Five Inventory—Versions 4a and 54. University of California, Berkeley, Institute of Personality and Social Research. (1991).

[68] Rammstedt B Danner D Bosnjak M. Acquiescence response styles: A multilevel model explaining individual-level and country-level differences. Pers Individ Dif (2013) 55:282–288. doi: 10.1016/j.paid.2013.03.020

[69] Lawton R Ashley L Dawson S. Employing an extended Theory of Planned Behaviour to predict breastfeeding intention, initiation, and maintenance in White British and South-Asian mothers living in Bradford. Br J Health Psychol. (2012) 17:854–871. 22950369 10.1111/j.2044-8287.2012.02083.x

[70] Naja F Chatila A Ayoub JJ Abbas N Mahmoud A, MINA collaborators. Prenatal breastfeeding knowledge, attitude and intention, and their associations with feeding practices during the first six months of life: a cohort study in Lebanon and Qatar. Int. Breastfeed. J. (2022) 17, 15. 35209913 10.1186/s13006-022-00456-x PMC8867651

[71] Srkalović Imširagić A Begić D Sarajlić Vuković I Rojnić Palavra I Orban M. Predictors of exclusive breastfeeding 6-9 weeks after delivery: a prospective cohort study public mental health perspective. Psychiatr. Danub. (2016) 28, 395–403. 27855431

[72] Dennis CL. Theoretical underpinnings of breastfeeding confidence: a self-efficacy framework. J Hum Lact (1999) 15:195–201. 10578797 10.1177/089033449901500303

